# European survey on neurosurgical management of primary central nervous system lymphomas and preoperative corticosteroid therapy

**DOI:** 10.1016/j.bas.2023.101791

**Published:** 2023-08-12

**Authors:** Florian Scheichel, Branko Popadic, Daniel Pinggera, Dariusz J. Jaskolski, Vincent Lubrano, Nicolas Foroglou, David Netuka, Bogdan Iliescu, Laszlo Novak, Camillo Sherif, Franz Marhold, Christian F. Freyschlag

**Affiliations:** a Karl Landsteiner University of Health Sciences, Krems, Austria; b Department of Neurosurgery, University Hospital St. Poelten, St. Poelten, Austria; c Department of Neurosurgery, Medical University of Innsbruck, Innsbruck, Austria; d Department of Neurosurgery and Neurooncology Medical University of Lodz, Norbert Barlicki University Hospital, Lodz, Poland; e Clinique de l'Union, Saint Jean, France; f ToNIC, Toulouse NeuroImaging Center, Université de Toulouse, Inserm, UPS, Toulouse, France; g Aristotle University of Thessaloniki, School of Medicine, Thessaloniki, Greece; h Central Military Hospital, Prague, Czech Republic; i Grigore T. Popa University of Medicine and Pharmacy, Iasi, Romania; j Department of Neurosurgery, Clinical Centre, University of Debrecen, Debrecen, Hungary

**Keywords:** Primary central nervous system lymphoma, Corticosteroid therapy, Survey

## Abstract

**Introduction:**

Preoperative corticosteroid therapy (CST) is common in primary central nervous system lymphoma (PCNSL) and may complicate histopathological diagnosis. There is an ongoing debate on the best management after preoperative CST.

**Research question:**

We aimed to survey how different European neurosurgical units treat PCNSL patients after preoperative CST.

**Methods:**

An English-language survey consisting of 21 questions addressing the management of patients with suspected PCNSL and preoperative CST was sent to European hospitals. The survey also included three clinical cases to assess the decision-making process in a clinical setting.

**Results:**

The survey was completed by 74 European hospitals. There was no clear consensus on how to treat a patient with PCNSL after CST. Accordingly, 24.3% responded that they would generally defer surgery regardless of a possible radiological response, 47.3% would defer surgery only if there is regression in preoperative MRI and the remaining 28.4% would defer surgery only if the tumor had completely vanished. Furthermore, there were distinct discrepancies in responses of neurosurgical units regarding their general management approach and their case-based decision in the three example cases. The results of our survey also showed regional differences and differences in treatment decisions between high-, intermediate- and low-volume centers.

**Discussion and conclusion:**

There was no clear consensus on how to treat patients with suspected PCNSL and preoperative CST. Furthermore, most centers also showed inconsistencies in their responses regarding their general approach as well as individual patient treatment. More high-quality evidence-based recommendations are needed to improve consensus and thus patient care.

## Highlights

• Half of centers postpone surgery for CNS lymphoma only on regression after steroids.
• There was no consensus on treatment of CNS lymphoma patients after steroid therapy.
• Individual treatment decisions differed distinctively in exemplary cases.

## 1 Introduction

Primary central nervous system lymphoma (PCNSL) is an aggressive and rare CNS pathology that accounts for approximately 3% of all primary brain tumors (Wöhrer et al., 2009). Therapy consists of rapid histopathological diagnosis followed by chemotherapy including systemic high-dose methotrexate (Ferreri et al., 2016; Hoang-Xuan et al., 2023). Neurosurgical management is mainly focused on obtaining tissue for histopathological diagnosis prior to definitive treatment. A common issue is preoperatively administered corticosteroid therapy (CST), which can lead to transient tumor shrinkage and potentially complicate histopathological diagnosis (Brück et al., 2013; Önder et al., 2015). The rate of inconclusive biopsies has been reported to be up to three times higher after preoperative CST (Scheichel et al., 2021). Therefore, if clinically possible, preoperative CST should be avoided until surgery has been performed (Hoang-Xuan et al., 2015; Fox et al., 2019). However, neurosurgeons are often confronted with the situation where PCNSL is highly suspected, and the patient has already received a substantial dose of CST. After an initial radiological response to CST, guidelines recommend tapering and pausing of CST until new radiological progression occurs before performing biopsy (Hoang-Xuan et al., 2015; Fox et al., 2019). However, the individual management of such patients after preoperative CST may vary significantly between centers as some recent studies described no reduction in diagnostic rates despite preoperative CST (Scheichel et al., 2021; Porter et al., 2008; Bullis et al., 2020).

Because of the conflicting data and the ongoing debate, we conducted an online survey to evaluate and highlight how patients with suspected PCNSL and preoperative CST are managed throughout Europe. Furthermore, we aimed to quantify how often neurosurgeons encounter this problem and how patient care could be improved.

## 2 Methods

### 2.1 Survey and study design

The authors designed an English-language survey consisting of 21 questions addressing the management of patients with suspected PCNSL and preoperative CST. The questionnaire was developed using the online tool Survey Monkey. Neuro-oncological specialists of 9 European countries (Austria, Czech Republic, France, Germany, Greece, Hungary, Netherlands, Poland, Romania) were asked to distribute the survey amongst their national neuro-oncology centers. The selection of possible participating countries was made considering the collaborative network of the Austrian authors and with the aim to achieve a good representation of Europe. Further, it was important to us to rule out duplicate responses and, accordingly, we did not want to address participating hospitals anonymously but through local distributors with a good local neuro-oncological network and good personal contacts. The survey was answered per center by the entire neuro-oncology team. All data was processed anonymously. The survey was available over a seven-month period from October 5th, 2020, to May 5th, 2021.

The countries were classified as European regions in Western (Austria, Germany, France, Netherlands), Southern (Greece) and Eastern Europe (Czech Republic, Hungary, Poland, Romania).

Classification was performed according to the Standard Classification for Countries and Territories of the United Nations Statistics Division.

### 2.2 Survey content

**Fig. 1:**
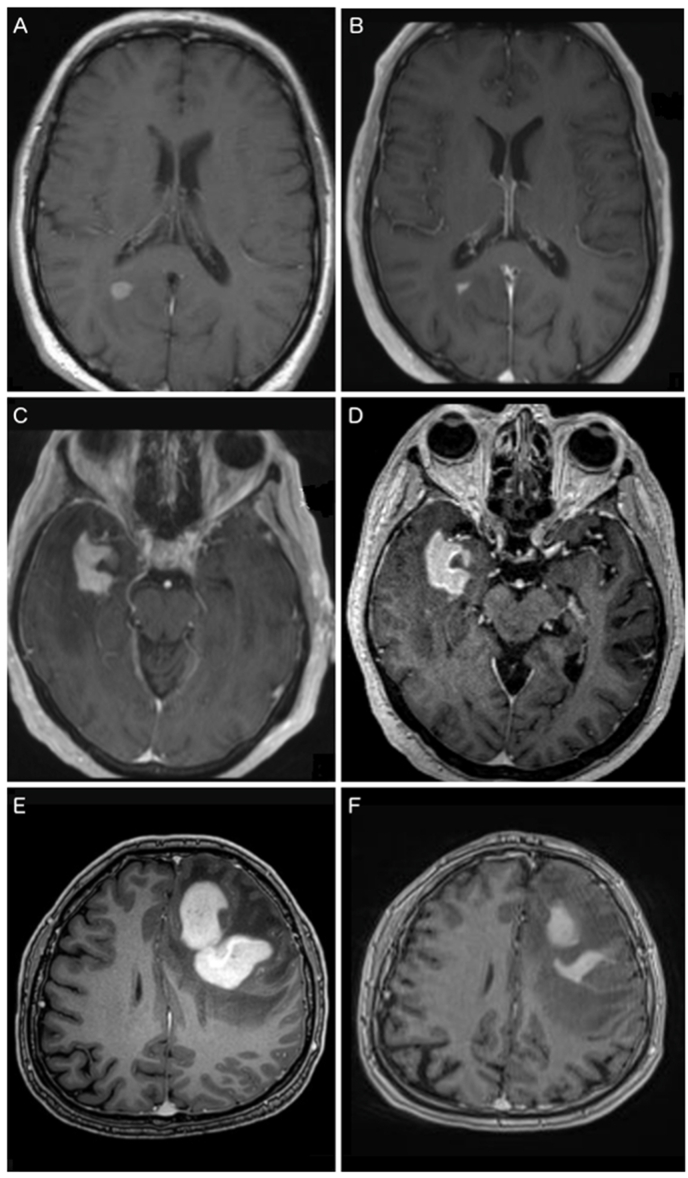
Three clinical cases. The first patient received 4 mg Dexamethasone 3 times a day for 7 days after the first MRI (A). The second MRI (B) was performed shortly before the planned biopsy 7 days after the first MRI. Corticosteroid treatment was still ongoing. The second patient received 4 mg Dexamethasone 3 times a day for 10 days between the two MRIs (C, D). Despite the corticosteroid therapy there was no regression on the follow-up MRI and surgery was scheduled for the following day. The third patient received 8 mg Dexamethasone 2 times a day for 7 days after the first MRI scan (E). The second MRI showed distinct regression (F). Corticosteroid treatment still was not tapered. Participating clinics were asked how each case would be treated at their institution.

The survey consisted of 21 questions divided into 3 sections. The first section aimed to collect anonymous data of the hospital and the neurosurgical department. The second section addressed the center's specific management of patients with suspected PCNSL and preoperative CST. This section was concluded by 3 illustrative cases with preoperative MRI sequences before and after administration of CST (Fig. 1). The final section included questions on surgical and diagnostic methods.

The complete survey can be found in the appendix.

### 2.3 Statistical analysis

Statistical analysis was performed using IBM SPSS Statistics for windows version 27 (V.27, version 27, SPSS Inc., IBM, Chicago, IL, USA). Given normal distribution (tested by Kolmogorow Smirnow test) metric data are described using mean and standard deviation. Skewed data are summarized using median and range. Categorical data are presented as absolute frequencies and percentages. To test for different distribution of nominal variables we used a Fisher exact test. A Mann Whitney *U* test was used to test for differences in median values of skewed metric data.

A p-value <0.05 was considered to indicate statistically significant results.

## 3 Results

### 3.1 Participating clinics

A total of 74 hospitals from 9 European countries (Table 1) participated in the survey. Most participants were from academic hospitals (74.3%) and the remaining from general hospitals (21.6%) or private clinics (4.1%). Almost all hospitals had a multidisciplinary neuro-oncological service (85.1%) and included PCNSL patients in their tumorboard (94.6%).

**Table 1:** Overview of geographical distribution of contributing centers and data on the general approach.

Parameter	Low volume	Intermediate volume	High volume	n	%
Hospitals	13 (17.6%)	39 (52.7%)	22 (29.7%)	74	100%
Austria	1 (9.1%))	9 (81.8%)	1 (9.1%)	11	14.9%
Czech Republic	1 (12.5%)	5 (62.5%)	2 (25%)	8	10.8%
France	1 (7.7%)	5 (38.5%)	7 (53.8%)	13	17.6%
Germany	0	3 (33.3%)	6 (66.7%)	9	12.2%
Greece	4 (44.4%)	4 (44.4%)	1 (11.1%)	9	12.2%
Hungary	1 (33.3%)	2 (66.7%)	0	3	4.1%
Netherlands	0	0	3 (100%)	3	4.1%
Poland	2 (15.4%)	9 (69.2%)	2 (15.4%)	13	17.6%
Romania	3 (60%)	2 (40%)	0	5	6.8%

General approach after CST					
Always delay surgery	2 (11.1%)	9 (50%)	7 (38.9%)	18	24.3%
Only if there was regression in MRI	6 (17.1%)	17 (48.6%)	12 (34.3%)	35	47.3%
Not if there is still an enhancing lesion for biopsy	5 (23.8%)	13 (61.9%)	3 (14.3%)	21	28.4%

Estimated patients with preoperative CST					41.5% (0 – 100)

Of all participating neurosurgical clinics, 29.7% were high-volume centers treating more than 10 PCNSL patients per year. Half of the participating centers estimated the number of PCNSL patients treated per year to be between 4 and 10 patients (52.7%), and 17.6% treated fewer than 4 patients per year.

The time between initial consultation of the neurosurgical department and the subsequent biopsy can be kept below one week in 43.2% of the participating clinics and between one and two weeks in 48.6%. Only 8.1% of the hospitals reported that the time to surgery usually exceeds 2 weeks. The median overall percentage of inconclusive biopsies in PCNSL patients reported by participating hospitals was 10% (range 0 – 70%, n = 72). There was a statistically significant difference in the distribution of estimated inconclusive biopsies on European regions with a median percentage of inconclusive biopsies in Western, Southern, and Eastern Europe of 10%, 20% and 25%, respectively (p = 0.001).

Furthermore, high-volume centers estimated their rate of inconclusive biopsies to be statistically significantly lower than intermediate-volume and low-volume centers with 10% (5 – 30%), 15% (1 – 70%) and 27.5% (0 – 55%), respectively (p = 0.007).

### 3.2 PCNSL patients and preoperative CST

The estimated median proportion of patients administered CST preoperatively was 41.5% (range 0-100).

Most participating departments are familiar with the most recent guidelines (71.6%) on PCNSL and 43.2% have established a standard protocol for managing PCNSL patients who were administered CST preoperatively. However, there was a significant discrepancy between low- and intermediate/high-volume centers, as the latter were more likely to be aware of current guidelines (69.2% vs 30.8%, p<0.001) and more likely to have a standard protocol for patients with preoperative CST (49.2% vs 15.4%, p = 0.032).

When asked how to treat a patient with suspected PCNSL that received preoperative CST, 24.3% responded that they would always defer surgery regardless of a possible radiological response. Almost half of the centers (47.3%) would defer surgery only if there is regression in preoperative MRI. The remaining 28.4% would perform biopsy despite regression if a target was still seen on preoperative MRI but would defer surgery if the tumor had completely vanished.

Related to the previous question, we asked how participating centers would define a radiological regression or response to CST. Most clinics compare structural MRI changes (47.3%) to define regression, 18.9% assess the maximal diameter of the target lesion, 25.7% perform volumetric analysis and 8.1% refer to the neuroradiologist's report.

**Fig. 2:**
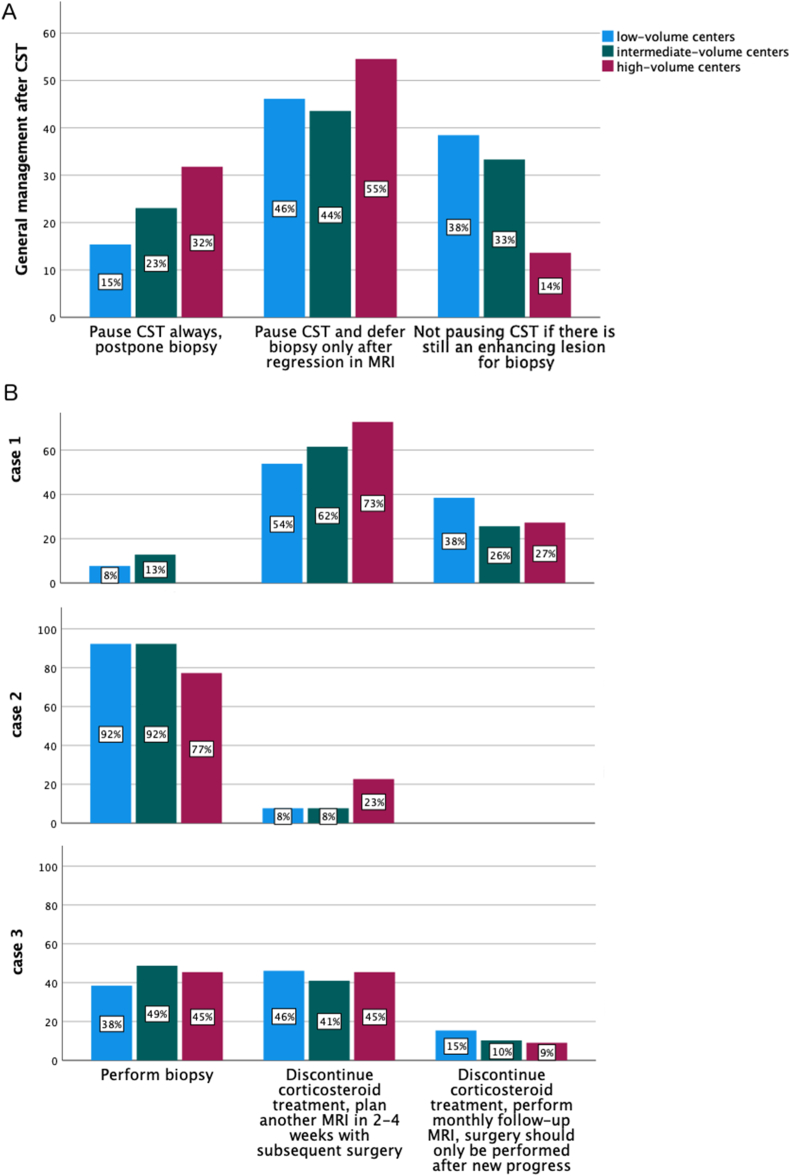
General approach on how to treat a patient with suspected PCNSL after preoperative CST for low-, intermediate- and high-volume centers in Europe. High-volume centers generally tended to pause CST more often than low-/intermediate-volume centers (A). There was significant discrepancy between low-, intermediate- and high-volume centers in the management of the exemplary cases and therefore no consensus either in or between the groups (B).

Of those clinics that would pause CST after regression, 44.9% would await re-progression before planning surgery and 55.1% would pause CST for a median of 10 days (range 4 – 30). High-volume centers tended to pause CST more often after radiological regression than low- and intermediate-volume centers combined with 86.4% and 65.4%, respectively (p = 0.067) (Fig. 2, A).

Of all participants, 78.4% stated that they think that preoperative CST lowers the diagnostic yield in PCNSL patients. Centers that did not believe that preoperative CST interferes with histopathological diagnosis tended to have a lower median rate of inconclusive biopsies with 10% compared to 15% (p = 0.063). 90.9% of all high-volume centers stated that they think that CST lowers the diagnostic yield, while only 74.4% and 69.2% of intermediate- and low-volume centers made this experience, respectively (p = 0.188).

Treatment of patients after CST did not differ significantly between centers that believe CST has a significant impact on diagnostic yield and the remaining clinics. Clinics that believe preoperative CST influences diagnostic yield would interrupt CST preoperatively in 24.1% (vs. 25%) regardless of regression, interrupt CST after regression in 50% (vs. 37.5%) and perform biopsy without interrupting CST in 25.9% (vs. 37.5%) if an enhancing lesion remained, respectively (p = 0.587).

#### 3.2.1 Illustrative cases

The first case (Fig. 1 A and B) of a PCNSL described a rather small target for biopsy which also showed distinct regression after CST. Only 8.1% of the clinics would perform biopsy without delay in this case. A pause of 2 – 4 weeks and subsequent MRI and surgery was the most common response (63.5%). The remaining clinics (28.4%) would perform monthly MRI until new progression is evident. Fig. 2, B shows the different answers for low-, intermediate- and high-volume centers. No high-volume center would opt for immediate surgery.

The lesion in the second case (Fig. 1 C and D) showed no response to CST. Almost all (87.8%) clinics would perform biopsy without delay and only 12.2% would wait 2 – 4 weeks. No center would wait until a new progression in serial MRIs.

The third case (Fig. 1 E and F) showed a patient who still had a promising target for biopsy despite significant regression of a large frontal lesion after CST. In this case 45.9% would proceed immediately to biopsy, 43.2% would wait 2-4 weeks and 10.8% would postpone surgery until re-progression.

**Fig. 3:**
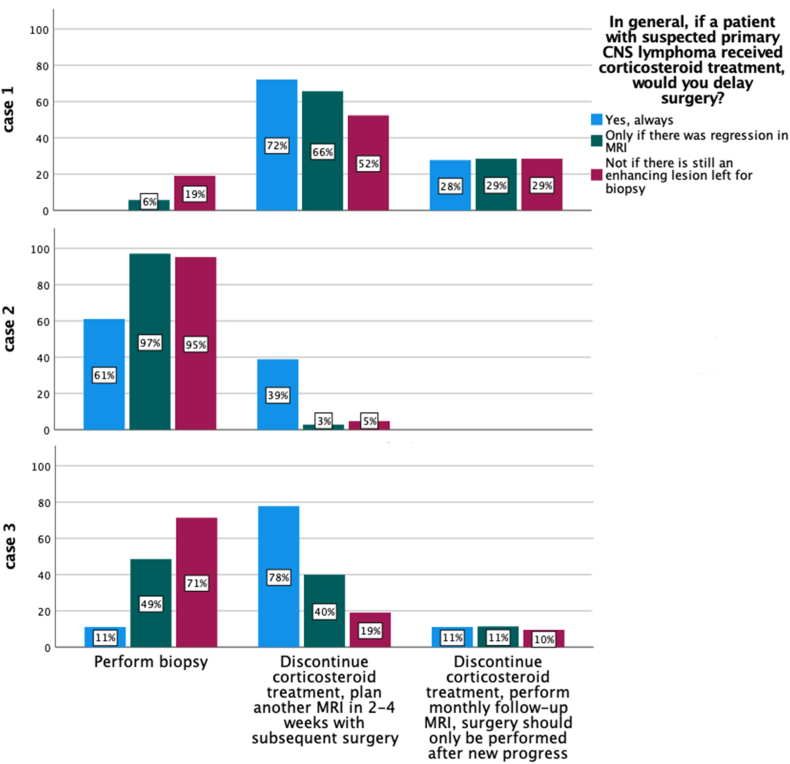
General treatment strategy and management of the clinical cases from Fig. 1. There was significant discrepancy between general approach and the treatment choices in the exemplary cases.

There was obvious discrepancy between the general management and specific treatment of the clinical cases as is illustrated in Fig. 3.

### 3.3 Technical questions

Preoperative lumbar puncture for cytology is routinely performed in 33.8%, in selected cases such as periventricular lesions in 39.2% and never performed in 27.0%.

We surveyed subjective opinion on the importance of lumbar puncture for PCNSL diagnosis. Only 23% of respondents reported that they experienced lumbar puncture to have significant diagnostic value in PCNSL, whereas 64.9% considered the diagnostic yield to be clinically insignificant.

Open surgery is considered by 75.7% of the participating clinics for suspected PCNSL in selected cases.

Most clinics (58.1%) do not use any additive technical aids in the operating room. Frozen section analysis is used by almost all remaining clinics (40.5%), 5-Aminolevulinic-acid by 20.3% and Sodium fluorescein (Yellow 560) by 2.7%.

## 4 Discussion

PCNSL is a rare CNS pathology and accounts for only 3% of all intracranial tumors (Wöhrer et al., 2009). Accordingly, 68.5% of the participating European neurosurgical clinics treat less than 10 PCNSL patients per year. This highlights the need for resilient data and guidelines for the management of this rare entity to enable efficient diagnosis and therapy. Currently, neurosurgeons should aim for a quick and definite histopathological diagnosis (Scheichel et al., 2022). The estimated time from consultation of neurosurgery to neurosurgical procedure was less than 2 weeks for most centers. This seems reasonable as real-world data showed a median delay from first symptoms to histopathological diagnosis between 35 and 47 days and a median time from first symptoms to neuroimaging of 15 days (Velasco et al., 2020; Houillier et al., 2020). Diagnosis of PCNSL is a multidisciplinary process that must be planned and executed efficiently and in coordination with colleagues from pathology, neurology, and oncology. Many additional staging examinations can be performed while the patient is awaiting surgery or histopathological results. Of these, lumbar puncture and cytology can potentially obviate the need for brain biopsy in rare cases (Hoang-Xuan et al., 2015), but our survey demonstrated that lumbar puncture is routinely performed in only one third of participants and in an additional third of cases with periventricular involvement of PCNSL. This might be explained by the low estimated diagnostic yield of lumbar puncture of our participants. Indeed, standard cytomorphologic analysis of cerebrospinal fluid is only diagnostic in 6 – 13.3% and therefore most patients ultimately need a biopsy (Morell et al., 2019; Hegde et al., 2005; Orfao et al., 2009; Schroers et al., 2010). However, if the diagnosis can be made by cytomorphologic analysis of CSF, no surgical intervention is required, thus avoiding the surgical risks to the patient. Since the time interval between initial radiological diagnosis and biopsy allows lumbar puncture in most cases without delaying surgical intervention, lumbar puncture should be strongly considered (Scheichel et al., 2022). In addition, the importance of lumbar puncture could increase significantly due to new diagnostic options such as liquid biopsies for the detection of MYD88 or CD79B mutations. (Hiemcke-Jiwa et al., 2018).

The current gold standard surgical procedure is stereotactic biopsy, as data on resection is limited with contradictory statements. In older studies patients with resections of an PCNSL performed worse, whereas recent data by Weller et al. found an improvement in overall and progression-free survival after resection of PCSNL compared to biopsy (Weller et al., 2012; Bataille et al., 2000). However, in their series surgery was more often performed for patients with a single lesion, leading to a potential bias. Only progression-free survival remained beneficial after adjusting for number of lesions. While 75.7% of all centers in our survey do consider open surgery for selected cases of suspected PCNSL, the standard surgical procedure is stereotactic biopsy. Participating centers estimated the median rate of inconclusive biopsies to be 10%. This is consistent with a recent retrospective study of 327 patients, which found that overall 9% of PCNSL patients needed a second biopsy (Velasco et al., 2020). Interestingly, high-volume centers estimated that they achieve a higher diagnostic yield than intermediate- and low-volume centers. The estimated diagnostic yield differed significantly between different European regions. While there could possibly be regional differences, this could also be just due to over or underestimation of inconclusive biopsies and cannot be clarified by our data.

Technical aids that could potentially increase the diagnostic yield are used by 41.9%. The most common tools are frozen section in 40.5% and 5-ALA in 20.3%. Both methods have been shown to reliably indicate whether diagnostic tissue has been obtained (Brainard et al., 1997; Kiesel et al., 2018). 5-ALA-fluorescence is positive in 79% - 83% and has a high positive predictive value for definitive diagnosis of almost 100% (Kiesel et al., 2018; Evers et al., 2017; Yamamoto et al., 2015). It therefore allows rapid adjustment of surgical strategy based on fluorescence status by providing feedback to the surgeon that in case of positive fluorescence diagnostic tissue has most likely been obtained.

### 4.1 Preoperative CST

Preoperative CST may lead to apoptosis of PCNSL tumor cells, resulting in transient shrinkage of the lesion (Sionov et al., 2008; Miller et al., 2005). This can complicate biopsy and make histopathological diagnosis more difficult (Brück et al., 2013; Önder et al., 2015). Participating hospitals reported that a median of 41.5% of their patients received CST preoperatively. This is less than the 59% of patients with CST in a recent literature review of 788 patients with PCNSL (Scheichel et al., 2021). Nevertheless, it indicates that approximately one in two patient requiring biopsy for PCNSL received CST preoperatively, highlighting the clinical importance of structured and evidence-based recommendations on this topic.

However, only 78.1% of participants reported that they felt that preoperative CST decreased the diagnostic yield of biopsy based on their clinical experience. This is interesting as a recent study and literature review found a threefold increased risk of inconclusive biopsies after preoperative CST (Scheichel et al., 2021).

Most recent guidelines addressing the challenges of preoperatively administered CST are the EANO guidelines on diagnosis and treatment of PCNSL, the guidelines of the British Society for Haematology and an evidence-based expert consensus statement of China (Hoang-Xuan et al., 2015; Fox et al., 2019; Chen et al., 2022). They all state that preoperative CST should be avoided if PCNSL is suspected due to the risk of inconclusive biopsies. In cases where preoperative CST has been administered, they advocate to pause CST prior to urgent biopsy. They argue that if there is no response or even progression after CST in preoperative MRI, there should be a relatively good probability of acquiring diagnostic tissue with biopsy ((Hoang-Xuan et al., 2015) – web appendix). However, after radiological regression, they recommend stopping CST, deferring biopsy and perform a follow-up MRI after approximately two weeks to plan urgent biopsy on regrowth.

Of all participants, those that stated that they are aware of recent guidelines, would more often perform a biopsy without delay after CST if there is still contrast enhancement with 31.1% compared to 19%, but the difference was not statistically significant (p = 0.392). We asked participants for their opinion on three specific cases in which preoperative CST was administered until the preoperative MRI. Case 1 (Fig. 1A-B) showed a patient with significant regression of a small PCNSL after CST. Accordingly, most clinics would defer biopsy (93.2%), probably because the estimated risk of obtaining inconclusive tissue was considered too high. Interestingly, 5.7% of the clinics that indicated that they would generally defer biopsy after CST in case of regression, opted for biopsy under CST in this particular case. On the other hand, 81% of the clinics that indicated they would generally perform biopsy without pausing CST if an enhancing lesion is remaining, opted to delay the biopsy (Fig. 3).

According to the guidelines, stopping CST, deferring biopsy and performing follow-up MRIs to detect regrowth would be the best management for this case.

Case 2 (Fig. 1C-D) described a patient with a large PCNSL lesion that was barely responsive after CST. Therefore, most participants (87%) would proceed with surgery without delay, even though this contradicts their reported general approach. Of all clinics that indicated that they would always delay surgery after CST, 61.1% would perform surgery immediately in this particular case (Fig. 3). According to guidelines, the chances of success of a biopsy in this case would be considered high.

The PCNSL lesion of the third patient (Fig. 1E-F) showed distinct regression after CST yet still a promising target for biopsy. In this case, 46.6% would proceed with biopsy without delay, 42.5% would wait 2-4 weeks and 11% would postpone surgery until new re-progression. This shows that the question of when to wait and when to perform biopsy after preoperative CST and tumor regression is far from clear in challenging cases. In addition, there was a significant discrepancy between participants’ responses regarding their general approach and the treatment of this case. Of the centers that indicated they would generally defer surgery or defer surgery in case of regression, 11.1% and 48.6%, respectively, would immediately perform a biopsy in this case. Contrary, of the clinics that indicated they would not postpone surgery if a contrast enhancing lesion was still present, 28.6% would defer surgery for this patient (Fig. 3). In this case, significant regression was observed after CST. Recent guidelines would therefore recommend discontinuing CST and postponing surgery until new progression in serial MRI (Hoang-Xuan et al., 2015). Yet, 45.3% of the clinics that are aware of guidelines would opt to perform surgery immediately in this case. While there are studies that found no significant difference in diagnostic yield with and without preoperative CST (Porter et al., 2008; Bullis et al., 2020; Binnahil et al., 2016), these studies are lacking the statistical power to draw that conclusion or suffering other limitations. Combined analysis of available literature showed a significant increased risk of inconclusive biopsies after CST (Scheichel et al., 2021). Nevertheless, no prospective data is available and there are no studies regarding the correlation of radiological response and diagnostic probability. The responses regarding the treatment of the three cases indicate that there is no consensus among European centers in the treatment of CST-pretreated PCNSL patients despite the available guidelines. The discrepancy between their general approach and the individual treatment of example cases further shows the complexity of this issue.

The results of our survey show the pronounced disagreement on the best neurosurgical management of corticosteroid pretreated PCNSL patients. This is important as it discloses that currently available guidelines have a negligible impact or are neglected possibly due to the low-level evidence they are based on. On top of that, our data not only demonstrated distinct interinstitutional differences but also remarkable inconsistencies in treatment decisions. These discrepancies in neurosurgical management of PCNSL patients concerning CST application show that a prospective trial would be sorely needed.

### 4.2 Limitations

The authors are aware of the limitations involved in surveys. To overcome the general criticism of survey studies, instead of sending the survey through national societies’ emails, we distributed the survey directly to neuro-oncology centers through one neurosurgeon per country.

## 5 Conclusion

The results of our Europe-wide survey demonstrate that there is no clear consensus on how to treat patients with suspected PCNSL and preoperative CST. In addition, most centers even showed inconsistencies in their own responses regarding their general approach and their case-based individual treatment approach. Therefore, more high-quality evidence-based recommendations are needed to improve consensus and thus patient care.

## Funding

The article processing charge was covered by the Open Access Publishing Fund of Karl Landsteiner University of Health Sciences, Krems, Austria.

## Author contributions

FS, BP, DP, FM, CF contributed to conception and design of the study and the survey. FS wrote the first draft of the manuscript. DJ, VL, NF, SN, BI, LN helped to distribute the survey among their national neuro-oncology centers. All authors contributed to manuscript revision, read, and approved the submitted version.

## Declaration of competing interest

The authors do not have any conflicts of interest, financial or otherwise.
