# Supplementary material for: European survey on neurosurgical management of primary central nervous system lymphomas and preoperative corticosteroid therapy
Source: Brain Spine. 2023 Aug 12;3:101791. doi: 10.1016/j.bas.2023.101791 (PMC10668066; doi:10.1016/j.bas.2023.101791)
Supplement: Multimedia component 1 [file mmc1.docx]

## European survey on neurosurgical management of primary central nervous system lymphomas and preoperative corticosteroid therapy

## Personal questions

1. What is your country of practice?
2. Where do you work?
   academic hospital / general hospital / private clinic
3. Do you have a multidisciplinary neurooncological service?
   yes / no
4. Does your tumorboard include patients with lymphomas?
   yes/no

## General questions

1. How many patients undergo biopsy or surgery for primary CNS lymphoma per year at your institution?
   1. 1-3
   2. 4-10
   3. >10
2. How many days pass usually between first consultation and biopsy of patients with suspected primary CNS lymphoma?
3. < 7 days
4. 7-14 days
5. >14 days
6. How many of your patients with suspected primary CNS lymphoma have already received corticosteroid therapy prior to consultation of your department?

_____ %

1. How often do you experience inconclusive biopsies in suspected primary CNS lymphoma?

____ %

## Questions about the handling of corticosteroid pretreated patients with suspected primary CNS lymphoma

1. Based on your clinical experience, do you think that corticosteroid pretreatment lowers the diagnostic yield in primary CNS lymphomas?
   1. Yes
   2. No
2. Are you aware of guidelines on handling CNS lymphoma (like for example the EANO guideline from the year 2015)?
3. Yes
4. No
5. Is there a defined standard procedure at your institution on how to handle patients with suspected primary CNS lymphoma that received corticosteroid therapy prior to biopsy?
6. Yes
7. No
8. In general, if a patient with suspected primary CNS lymphoma received corticosteroid treatment, would you delay surgery?
9. Yes, always
10. Only if there was regression in MRI
11. Not if there is still an enhancing lesion left for biopsy
12. How do/would you define a radiological regression in patients with suspected primary CNS lymphoma after corticosteroid treatment?
13. Comparison of structural MRI changes
14. Maximum diameter of contrast enhancement
15. Volumetric analysis of contrast enhancement
16. Report of the neuroradiologist
17. In general, if a patient with suspected primary CNS lymphoma received corticosteroid treatment, how long would you delay surgery after tapering of corticosteroid treatment?
18. Not at all
19. Yes: _____days
20. Yes, until new progression
21. The following patient received 4mg Dexamethasone 3 times a day for 7 days after the first MRI (A). The second MRI (B) was performed shortly before the planned biopsy 7 days after the first MRI. Corticosteroid treatment was still ongoing. How would this case be handled at your institution?


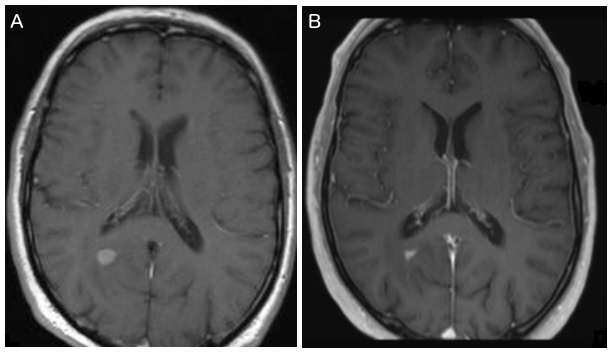


1. Perform biopsy
2. Discontinue corticosteroid treatment, plan another MRI in 2-4 weeks with subsequent surgery
3. Discontinue corticosteroid treatment, perform monthly follow-up MRI, surgery should only be performed after new progression
4. The following patient received 4mg Dexamethasone 3 times a day for 10 days between the two MRIs shown below (A, B). Despite the corticosteroid therapy there was no regression on the follow-up MRI (B). The surgery was planned for the day of the second MRI (B). Corticosteroid therapy was still ongoing. How would you handle this case at your institution?


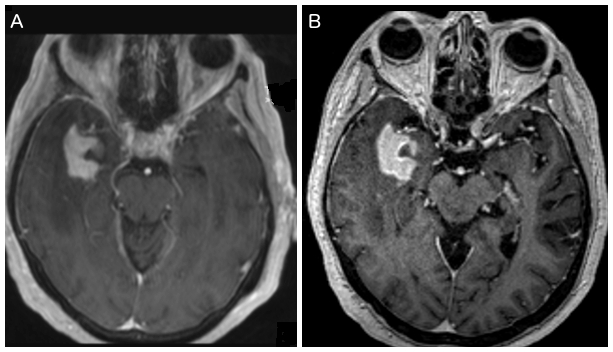


1. Perform biopsy
2. Discontinue corticosteroid treatment, plan another MRI in 2-4 weeks with subsequent surgery
3. Discontinue corticosteroid treatment, perform monthly follow-up MRI, surgery should only be performed after new progression
4. The following patient received 8mg Dexamethasone 2 times a day for 7 days after the first MRI scan (A). The second MRI showed distinct regression. Corticosteroid treatment still was not tapered. How would this case be handled at your institution?


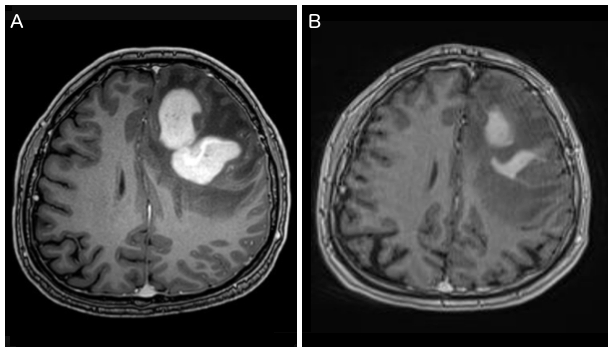


1. Perform biopsy
2. Discontinue corticosteroid treatment, plan another MRI in 2-4 weeks with subsequent surgery
3. Discontinue corticosteroid treatment, perform monthly follow-up MRI, surgery should only be performed after new progression
4. Do you consider open surgery for biopsy in patients with an accessible lesion after inconclusive stereotactic biopsy?
5. Yes
6. No

## Questions on biopsy technique

1. What additive surgical/In-OR technique do you use for biopsy of suspected primary CNS lymphoma? (multiple choice)
2. none
3. frozen section
4. 5-ALA Fluorescence
5. Yellow560 Fluorescence
6. Do you usually perform lumbar puncture in patients with suspected primary CNS lymphoma?
7. Yes, routinely
8. only in selected cases (with periventricular involvement)
9. no
10. Based on your clinical experience what do you estimate the diagnostic yield of lumbar puncture in primary CNS lymphomas at your institution?
